# Artificial Intelligence-Assisted Detection of Canine Impaction, Localization, and Classification from Panoramic Images: A Diagnostic Accuracy Comparative Study with CBCT

**DOI:** 10.3390/children13040507

**Published:** 2026-04-04

**Authors:** Narmin M. Helal, Abdulrahman F. Aljehani, Sawsan A. Alomari, Reem A. Mahmoud, Hanadi M. Khalifa

**Affiliations:** 1 Department of Pediatric Dentistry, Faculty of Dentistry, King Abdulaziz University, Jeddah 21589, Saudi Arabia; abdulrahmanalj19@gmail.com (A.F.A.); dr.saalomari@gmail.com (S.A.A.); reem19.mahmoud@gmail.com (R.A.M.); 2 Oral Radiology Division, Oral Diagnostic Sciences Department, Faculty of Dentistry, King Abdulaziz University, Jeddah 21589, Saudi Arabia; hmkhalifah@kau.edu.sa

**Keywords:** maxillary canine impaction, panoramic radiography, cone-beam computed tomography, deep learning, artificial intelligence

## Abstract

**Background/Objectives:** This study aimed to develop and evaluate deep learning models for the detection, localization, and classification of impacted maxillary canines, and to compare their performance with cone-beam computed tomography (CBCT) as the reference standard. **Methods:** This cross-sectional diagnostic accuracy study was conducted at King Abdulaziz University Dental Hospital to develop and validate artificial intelligence (AI) models for detecting and localizing maxillary canine impactions using panoramic and cone-beam computed tomography (CBCT) imaging data. A total of 641 panoramic ra and 158 CBCT scans were collected, of which 158 cases had matched panoramic–CBCT pairs for localization analysis. Images were annotated and validated by expert radiologists and orthodontists, with consensus review ensuring labeling reliability. Data augmentation expanded each panoramic and CBCT category to 500 samples for panoramic and 1000 samples for CBCT, resulting in 1935 panoramic and 5703 CBCT images after preprocessing and normalization. The datasets were divided into (training + validation) (80%) and testing (20%) subsets. MobileNetV2 architectures were used for classification, and whdiographsile, a ResNet-50–based Few-Shot Learning framework, enabled spatial localization of impacted canines. Models were trained using the Adam optimizer with a learning rate of 1 × 10^−4^ and evaluated using accuracy, precision, recall, F1 score, and area under the receiver operating characteristic curve (AUC). Cohen’s kappa and 95% confidence intervals were used to assess agreement between AI predictions and expert annotations. **Results:** Panoramic classification achieved 94% accuracy, demonstrating the highest performance in normal cases and reduced recall for bilateral impactions. The CBCT classifier achieved 98% accuracy across positional categories. Cross-modality prediction reached 93.5% accuracy, with strong agreement compared to CBCT (Cohen’s kappa = 0.91). Expert review confirmed reliable localization of impacted canines on both imaging modalities. **Conclusions:** Artificial intelligence applied to panoramic radiographs supports the detection, localization, and characterization of impacted maxillary canines with performance comparable to CBCT. This approach may enable lower-radiation decision support for clinical triage.

## 1. Introduction

Tooth impaction refers to a tooth remaining below the bone after its expected eruption period or failing to erupt into the oral cavity. Impaction occurs when normal tooth development is disrupted [1]. Terms like delayed eruption, primary retention, submerged teeth, and impacted teeth are common in clinical practice to describe this condition. A canine is impacted if the opposing canine has erupted for at least six months with complete root development or if eruption is halted after full root development [2]. Canine impaction can affect maxillary and mandibular arch length, cause crowding, and impact occlusion, which may influence orthodontic treatment planning [3].

In Saudi Arabia, canine impaction is a significant dental concern, with regional studies reporting varying prevalence rates. The Eastern Province reported a high incidence, with maxillary canines comprising most impacted teeth in a sample of 539 patients [4]. Studies from the western and southwestern regions found prevalence rates of 4% and 5.35%, respectively, based on large retrospective radiographic reviews [5,6]. Maxillary canine impaction poses a significant challenge in orthodontics and oral surgery, with prevalence varying globally from 1.1% in Sweden, where interceptive protocols exist, to 6.8% in Iraq [7,8]. Females are more affected, with ratios from 1.5:1 to 2:1 [9]. Palatal impaction is more common (72.6%) than buccal, and bilateral cases occur in 22.3% [9,10]. Early detection is essential in the management of canine impaction, as multiple studies highlight its importance in reducing complications, enabling timely intervention, and minimizing the need for complex treatments. Several sources indicate that early diagnosis facilitates effective interceptive measures, which can prevent complete impaction or promote appropriate eruption pathways [11,12].

Panoramic radiographs are integral to the diagnosis of canine impaction, as multiple studies have demonstrated their effectiveness in identifying impacted canines and predicting related complications. Several investigations highlight their utility in localizing impacted teeth, particularly palatally impacted canines, while indicating that additional imaging may be necessary for accurate buccal localization [13,14]. Other research supports the diagnostic reliability of panoramic radiographs, reporting strong concordance with alternative radiographic methods and confirming their value in predicting ectopic eruption and facilitating early assessment in pediatric and adolescent populations [15]. In addition to detection, panoramic X-rays provide critical information for classifying impactions and informing surgical intervention decisions, with automated classification tools further supporting clinical decision-making [16]. Furthermore, panoramic radiographs have been shown to predict root resorption, thereby assisting clinicians in determining when supplemental CBCT imaging is appropriate [17].

The Ericson and Kurol sector classification has shown moderate reliability as a predictor of canine impaction. It categorizes the position of the maxillary canine relative to adjacent incisors on panoramic radiographs, with greater sector overlap linked to a higher likelihood of impaction. Research indicates that canines overlapping the lateral incisor midline are associated with a significantly increased risk of impaction, with an estimated probability of 87% [18]. Nevertheless, studies have identified substantial limitations in manual measurement techniques [19]. A systematic review by Ravi et al. [20] found that various angular radiographic measurements may help predict impaction risk, although considerable variability exists across studies. Specifically, angles greater than 19.9° between the canine’s long axis and the midline, or 20.01° between the canine and lateral incisor, are linked to a higher probability of impaction. Additional evidence suggests that traditional two-dimensional measurements can produce inconsistent results, indicating that three-dimensional imaging techniques may provide a more reliable basis for classification [21].

Artificial intelligence (AI) exhibits significant potential in the analysis of panoramic radiographs across diverse diagnostic domains, consistently achieving high performance metrics in various medical applications. Deep learning models, especially convolutional neural networks (CNNs), are now integral to contemporary dental diagnostics, with approximately 63.5% of related studies emphasizing diagnostic applications [22]. While initial research did not always comprehensively report their effectiveness in orthodontic radiographic imaging, a growing body of evidence indicates that CNNs reliably detect dental pathologies and efficiently segment and categorize teeth, as demonstrated in several systematic reviews [23]. CNN-based methods have demonstrated substantial effectiveness in improving diagnostic accuracy and workflow efficiency within orthodontics. Several reviews indicate that these approaches achieve accuracy rates exceeding 90% for key clinical tasks, such as automated landmark detection, tooth segmentation, and defect classification. These methods frequently surpass manual assessments and significantly decrease examination time [24,25]. Furthermore, classification models have attained over 98% accuracy in differentiating X-ray images with and without teeth, thereby facilitating more effective orthodontic treatment planning [26]. Large-scale applications further demonstrate the robustness of these systems. One study, which utilized 61,842 orthodontic diagnostic images and was validated on 13,729 new samples, achieved a 99.24% accuracy rate. This approach also demonstrated faster processing and greater emphasis on clinically meaningful image features [27].

Although AI is increasingly used in dental radiology, major gaps remain in assessing impacted maxillary canines. Previous research mostly detected canine impaction or predicted related complications, such as root resorption, but did not precisely classify or localize impactions. Many AI models use pre-trained architectures on small or unrepresentative datasets, which may not reflect real clinical cases or local populations. In Saudi Arabia, there are no locally developed and validated AI models that combine panoramic radiographs and CBCT to identify, localize, and classify impacted maxillary canines. CBCT is the diagnostic gold standard, but its higher radiation dose and cost limit routine use. There is little evidence on whether AI analysis of panoramic radiographs can match CBCT’s diagnostic performance.

This study aims to develop and evaluate an AI-based deep learning framework for automatically detecting, localizing, and classifying impacted maxillary canines on panoramic radiographs, using CBCT as the reference standard. This system will support early diagnosis, aid clinical decisions, and improve the safety and efficiency of dental workflows.

## 2. Materials and Methods

### 2.1. Study Protocol and Ethical Approval

This study was conducted at King Abdulaziz University Dental Hospital in Jeddah, Saudi Arabia. It aimed to develop and validate AI-based models for detecting and localizing maxillary canine impactions using panoramic and CBCT imaging data. Additionally, all procedures adhered to the Declaration of Helsinki and institutional ethical standards. The study was approved by the institutional review board (IRB No. 13-01-25).

### 2.2. Data Acquisition and Image Sources

**Figure 1:**
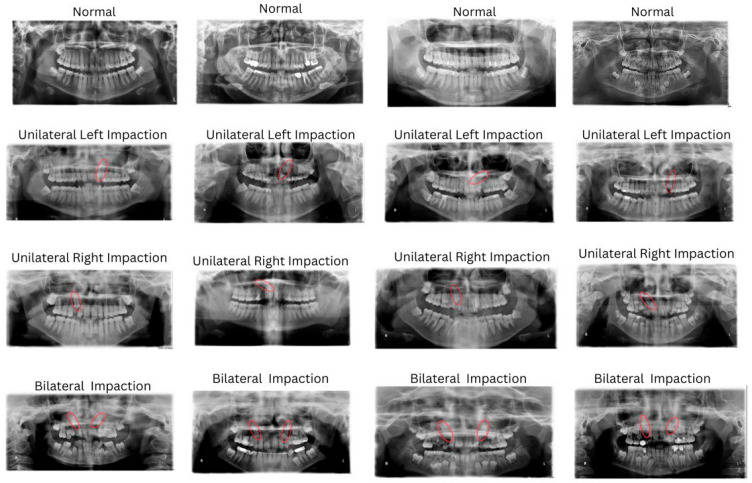
Panoramic canine impaction annotation sample set. Red circles indicate the location of the impacted maxillary canines on the panoramic radiographs.

A total of 641 anonymized panoramic radiographs were collected from patient records at King Abdulaziz University dental hospital (Figure 1). Images were categorized as 341 normal images (no impaction), 191 bilateral impactions (both canines), 53 unilateral left impactions, and 56 unilateral right impactions. Further, 158 panoramic images had corresponding CBCT scans, forming a matched subset used for 3D localization model training and validation. The panoramic dataset was used for classification modeling, while the matched CBCT subset was utilized for impaction localization and orientation assessment.

### 2.3. Inclusion and Exclusion Criteria

The inclusion criteria comprised patients with available digital panoramic radiographs of diagnostic quality, showing either normal maxillary canine eruption or radiographically confirmed impaction evaluated by an expert radiologist. Exclusion criteria involved cases with congenital craniofacial anomalies, pathological lesions (e.g., cystic or neoplastic changes), or image artifacts that could obscure anatomical landmarks.

### 2.4. Image Annotation and Expert Validation

All images were independently evaluated and annotated by two calibrated clinicians for impaction types and localization categories. Inter-observer agreement between annotators was assessed using Cohen’s kappa coefficient prior to model training. In instances of diagnostic disagreement, a qualified orthodontist served as an expert reviewer to determine the final label. Subsequently, all annotations were subjected to cross-validation by an experienced oral and maxillofacial radiologist to confirm consistency and accuracy. CBCT images served as the gold standard for confirming the presence and position of impacted maxillary canines. Each panoramic radiograph was annotated for the presence or absence of impacted maxillary canines, also the localization of the impaction as buccal, palatal, or central, in addition to the determination of canine overlap (Figure 1). The annotations were performed using makesense.ai, a web-based annotation tool. Images were labelled and exported in YOLO format for compatibility with object detection models. All data were anonymized prior to analysis.

### 2.5. Data Preprocessing and Augmentation

#### 2.5.1. Panoramic Radiographs

**Figure 2:**
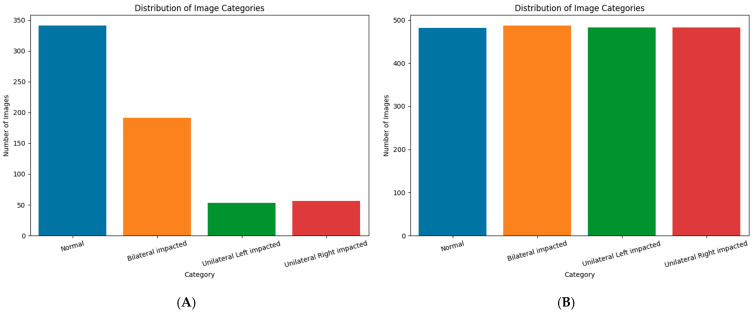
Class distribution of panoramic radiographs (**A**) before augmentation and (**B**) after augmentation, showing improved balance across normal, bilateral, and unilateral canine impaction categories.

High-resolution panoramic X-ray images were curated from clinical archives and annotated according to canine impaction status. Each class (normal, bilateral impaction, unilateral left, and unilateral right) was augmented to 500 samples to achieve class balance and improve model generalizability. The preprocessing and augmentation steps per image included the following: (1) conversion to RGB and resizing to 224 × 224 pixels for model compatibility; (2) pixel normalization to the [0, 1] range; and (3) augmentation using random rotations (±20°), horizontal shifts (10%), zooms (±20%), and horizontal flips to simulate patient positioning and imaging variability. This process yielded a balanced panoramic dataset of approximately 1935 images after quality control (Figure 2).

#### 2.5.2. CBCT Volumes

**Figure 3:**
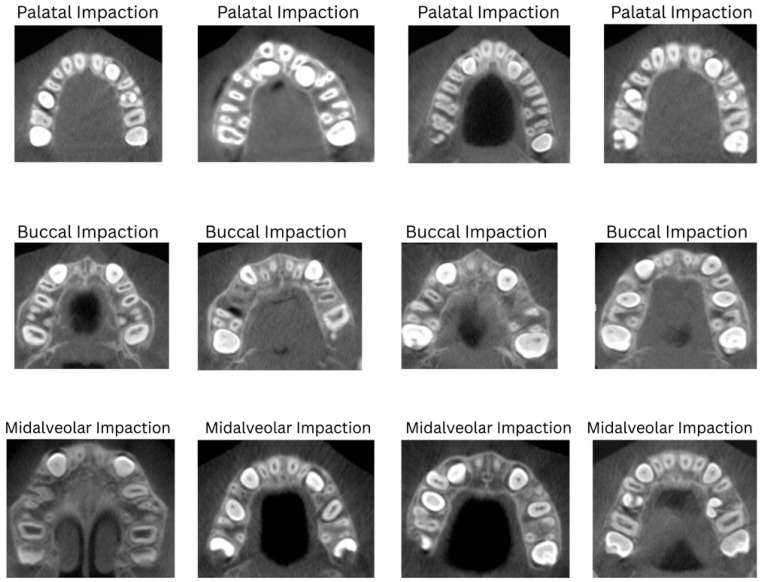
CBCT for impaction spatial information sample set.

**Figure 4:**
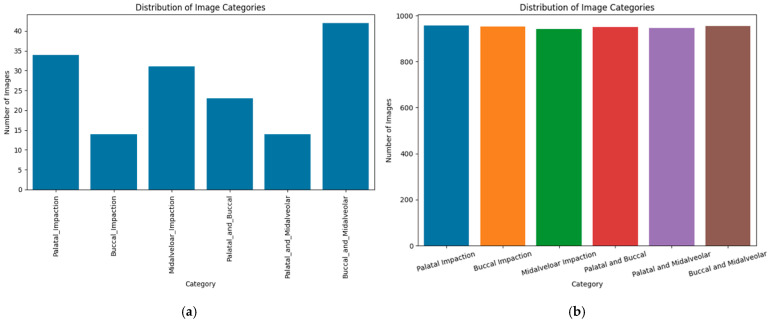
Distribution of CBCT images by impaction subtype: (**a**) original dataset before augmentation and (**b**) balanced dataset after augmentation to 1000 volumes per class.

CBCT datasets were preprocessed and augmented using a parallel approach tailored for 3D volumetric data. Each of the six impaction subtypes (palatal, buccal, midalveolar, palatal–buccal, palatal–midalveolar, and buccal–midalveolar) was expanded to 1000 scans to achieve uniform class distribution. The processing steps included: (1) voxel intensity normalization to standardize grayscale values across scanners; (2) Resampling all volumes to a consistent isotropic voxel size (e.g., 1 mm^3^); (3) three-dimensional augmentations: random rotations (±15°), translations (up to 10%), isotropic scaling (±10%), and left–right flipping (where anatomically appropriate); and (4) intensity perturbation using contrast adjustment and Gaussian noise to simulate scanner variability. All augmented volumes were visually inspected to ensure anatomical plausibility and correct label preservation, resulting in a balanced CBCT dataset of approximately 5703 volumes after quality control (Figure 3 and Figure 4).

### 2.6. Model Architecture and Training

#### 2.6.1. Classification Models

**Figure 5:**
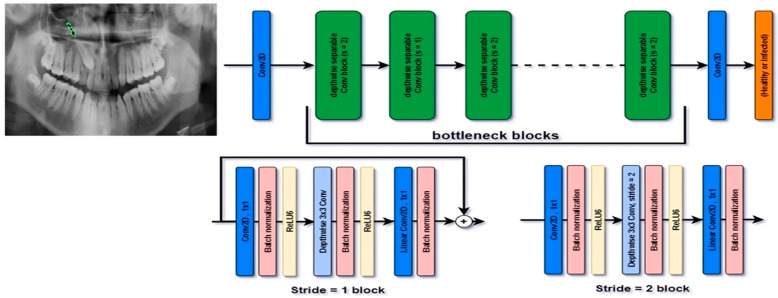
MobileNetV2 architecture. Green Arrow highlights the location of the impacted maxillary canine on the panoramic radiograph.

Two CNN–based models were developed to classify images from panoramic and CBCT datasets. MobileNetV2 was selected as the backbone network due to its computational efficiency and robust performance on limited medical datasets (Figure 5). The network was initialized with ImageNet pre-trained weights, and transfer learning was applied by fine-tuning the final layers to accommodate the specific number of output classes for each imaging modality. Additionally, the model classified the panoramic images into four categories: normal, bilateral impaction, unilateral left impaction, and unilateral right impaction. Additionally, the model was trained to classify CBCT scans into six impaction categories: palatal, buccal, midalveolar, palatal–buccal, palatal–midalveolar, and buccal–midalveolar. Data augmentation, as described in earlier sections, was applied to mitigate class imbalance and enhance the generalizability of both models.

#### 2.6.2. Localization Models

To achieve precise spatial identification of impacted maxillary canines, a localization model was developed utilizing a Few-Shot Learning (FSL) framework, specifically selected for its efficacy with limited annotated datasets. The architecture utilized ResNet-50 as the feature-extraction backbone, implemented via the PyTorch library version: 3.13.0. This FSL strategy enabled the model to infer complex spatial relationships and recognize impaction patterns from sparse labeled data, a significant advantage for identifying rare or anatomically intricate impaction cases. By analyzing the radiographic context, the network delineated the position and orientation of the impacted tooth relative to adjacent anatomical landmarks, facilitating integrated spatial localization and categorical classification. Additionally, localization performance was evaluated using a custom IoU-based accuracy metric. Predictions with confidence scores ≥ 0.5 were retained. A detection was considered correct if it achieved an IoU ≥ 0.5 with the corresponding ground-truth annotation and matched the assigned class label.

#### 2.6.3. Training and Evaluation Strategy

The dataset was randomly partitioned into (training + validation) (80%) and testing subsets (20%). The split was stratified by class to maintain proportional representation across impact categories. All image augmentations were applied exclusively to the training set to prevent data leakage. The validation subset was used for model tuning and early stopping, while the independent test set was reserved for final performance evaluation (Table 1). To prevent information leakage and ensure unbiased data evaluation, each patient contributed images exclusively to one subset only (training, validation, or testing). No images from the same patient appeared in more than one subset. All image augmentations were applied primarily to the training set to enhance model robustness. Augmented versions of test images were generated only after dataset splitting and were used for robustness analysis; however, primary model evaluation was conducted exclusively on original, non-augmented test images. Both classification and localization models were trained under uniform experimental conditions to ensure comparability. Training was performed on a workstation equipped with an NVIDIA GPU (GeForce RTX 4050). The training parameters included the use of the PyTorch framework and the Adam optimizer with an adaptive learning rate. The loss function employed was cross-entropy loss for multi-class classification tasks. Batch sizes were set to 32 for panoramic images and 8 for CBCT scans. The models were trained for 15 epochs, with early stopping after five epochs without improvement. The learning rate was initially set to 1 × 10^−4^ and decayed by 10% upon reaching a plateau. Input images were resized to 224 × 224 pixels across both modalities, with the dataset split stratified into training and validation sets at an 80:20 ratio.

### 2.7. Statistical and Diagnostic Accuracy Analysis

This study was designed as an image-based diagnostic accuracy study; therefore, the unit of analysis was the radiographic image rather than the patient. All statistical analyses were conducted using Python (v3.10) and SPSS (v27). To evaluate diagnostic reliability, the AI model’s outputs were compared to expert-labeled reference standards. Model performance was evaluated through metrics including accuracy, precision, recall (sensitivity), specificity, and F1 score, with macro-averaged F1 and AUC reported for multi-class problems. Confusion matrices were generated to visualize performance on individual classes. Cohen’s kappa coefficient (κ) was calculated to assess the agreement between AI predictions and expert annotations. Additionally, 95% confidence intervals (CIs) were computed for each performance metric, and a significance threshold of *p* < 0.05 was adopted to determine statistical significance. Prior to model comparison, inter-rater agreement among human experts was analyzed to establish a baseline for diagnostic reproducibility using Cohen’s kappa (κ) statistic, where κ values above 0.75 indicate substantial agreement. In cases of disagreement, a third senior radiologist adjudicated the final label, which served as the reference standard for AI training and evaluation.

## 3. Results

### 3.1. Dataset Characteristics

The independent test dataset included 387 panoramic radiographs and 1140 CBCT volumes, showing a wide range of maxillary canine eruption patterns seen in clinical practice. The panoramic images featured normally erupted canines as well as cases with unilateral and bilateral impactions, reflecting common diagnostic scenarios encountered in routine orthodontic work. Both left- and right-sided unilateral impactions were present, along with cases involving both sides. The CBCT test subset consisted of scans with confirmed impacted maxillary canines in various positions, including palatal, buccal, midalveolar, and combined locations. CBCT imaging was used as the reference standard to confirm impaction presence and three-dimensional positioning. The preserved case distribution in the independent test dataset allowed evaluation of classification and localization performance across clinically relevant impaction types while reflecting realistic diagnostic prevalence. Performance metrics reported in this study are based on the independent set of original test images. (Table 2).

### 3.2. Inter-Rater Agreement

Inter-rater agreement between the two expert annotators demonstrated substantial reliability (Cohen’s κ = 0.81; 95% CI, 0.76–0.86) for panoramic classifications and κ = 0.78 (95% CI, 0.72–0.84) for CBCT localization, supporting the consistency of the reference annotations. All diagnostic discrepancies (*n* = 23) were resolved through consensus with a third senior radiologist, resulting in the final reference standard used for model evaluation, which confirmed the reliability of the labeling process.

### 3.3. Panoramic Image Classification

**Figure 6:**
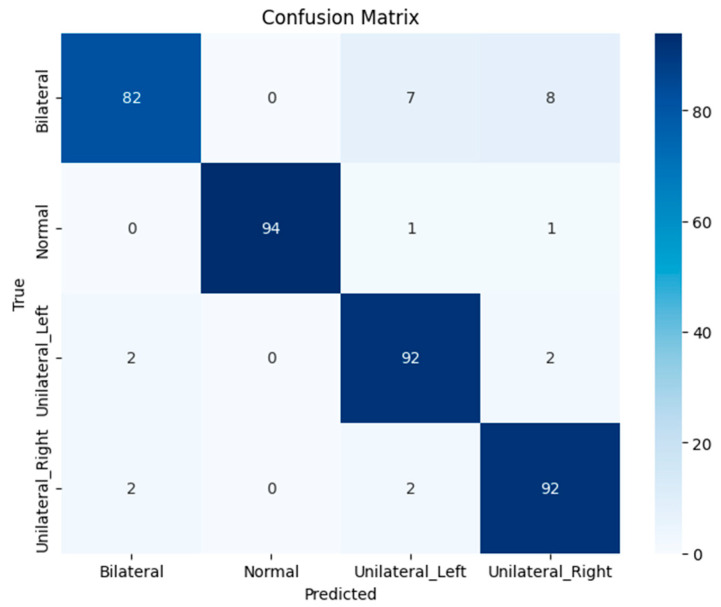
Confusion matrix of the MobileNetV2 panoramic classification model, showing class-wise prediction performance across four impact categories.

The panoramic image model demonstrated an overall accuracy of 94%. Among the evaluated groups, the normal cases achieved the highest performance, with a precision of 0.99, a recall of 0.98, and an F1-score of 0.99 (*n* = 96). Conversely, bilateral impaction cases showed the lowest performance, with a precision of 0.95, a recall of 0.85, and an F1-score of 0.90 (*n* = 97). The unilateral left and unilateral right groups also achieved strong results, further highlighting the model’s effectiveness across categories. Additional metrics and detailed results are presented in Table 3, while Figure 6 displays the confusion matrix.

### 3.4. CBCT Image Classification

**Figure 7:**
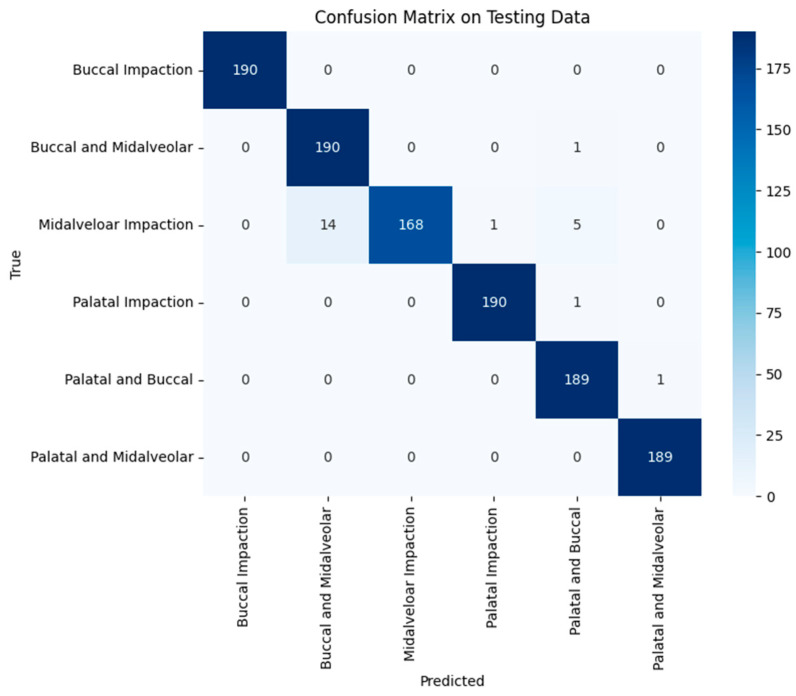
Confusion matrix of the MobileNetV2-based model for CBCT classification of impacted maxillary canine positions.

The CBCT-based model achieved 98% accuracy, demonstrating a clear ability to distinguish among impaction patterns. When comparing groups, palatal impaction cases (*n* = 191) had a precision and recall of 0.99, while midalveolar cases (*n* = 188) had a precision of 0.97 and a recall of 0.89. Across all categories, precision and recall ranged from 0.93 to 0.99. Table 4 summarizes these results, and Figure 7 presents the confusion matrix.

### 3.5. Localization Results

**Figure 8:**
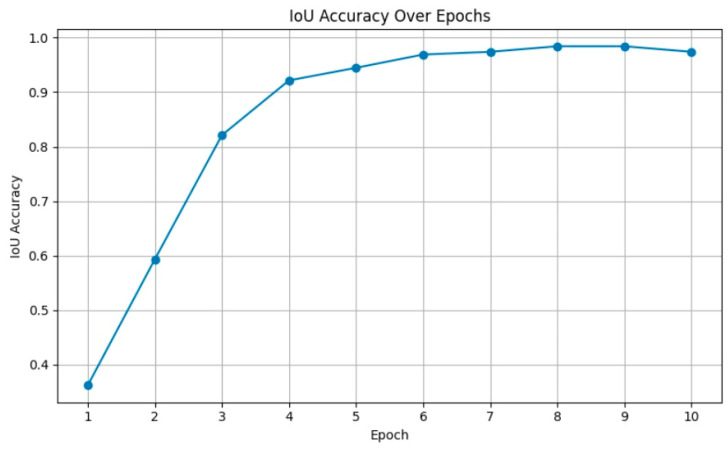
IoU-based localization accuracy over training epochs (IoU ≥ 0.5, confidence ≥ 0.5).

**Figure 9:**
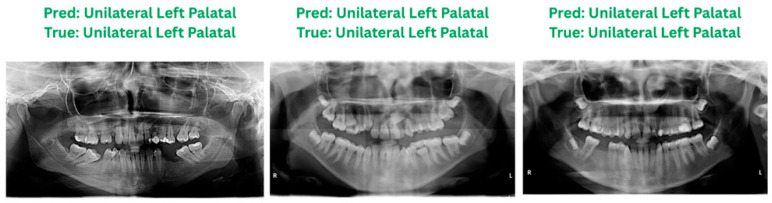
DenseNet121 model prediction on unseen data.

Localization differs from classification, as it is evaluated by comparing predicted bounding boxes to expert-annotated ground truth. Due to the scarcity of fully annotated localization datasets, expert assessment and visual comparison were used in this study. The Few-Shot Learning model, built with ResNet-50 and Faster R-CNN, reliably identified impacted canine teeth in both panoramic and CBCT images. As shown in Figure 8, the localization model achieved a final IoU-based accuracy of 98.8%, demonstrating high spatial agreement between the predicted and reference bounding boxes. These results suggest the model’s potential in clinical settings, especially dental diagnostic software. Figure 9 shows sample labelling and localizations.

### 3.6. Cross-Modality Impaction Prediction

**Figure A1:**
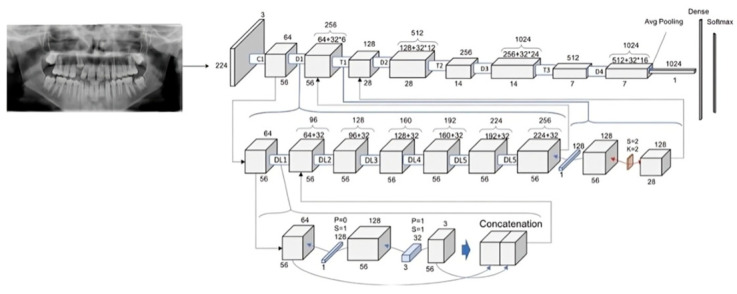
DenseNet-121 architecture illustrating dense blocks with a feature map concatenation, transition layers with pooling, and the final global average pooling and SoftMax layers used for image classification.

Using paired panoramic and CBCT data, the DenseNet121 architecture (Figure A1) was employed to classify six CBCT-based categories solely from panoramic images. Qualitative cross-modality prediction results on unseen panoramic images are illustrated in Figure 9. To ensure balanced representation, data augmentation was employed so each class contained 500 images for panoramic and 1000 images for CBCT. The AI model’s predictions from panoramic images achieved an overall accuracy of 93.5% compared to the CBCT ground truth. Furthermore, a Cohen’s kappa coefficient of 0.91 indicates excellent agreement, suggesting that the model’s panoramic predictions closely align with CBCT assessments.

## 4. Discussion

This study demonstrates the ability of deep learning models to accurately detect and localize maxillary canine impactions using both panoramic and CBCT imaging. The classification framework based on MobileNetV2 demonstrated high diagnostic accuracy, achieving 94% with panoramic radiographs and 98% with CBCT scans, effectively differentiating between normal and various impaction types. Additionally, the localization model, utilizing Few-Shot Learning and ResNet-50 architectures, successfully identified impacted regions, producing bounding boxes that closely aligned with expert annotations. These results underscore the potential of integrating AI-driven diagnostic tools into dental imaging workflows, enabling consistent and objective interpretation of complex impaction patterns. The high level of agreement between AI predictions and expert evaluations (κ = 0.91) further underscores these models’ ability to support clinical decision-making and improve diagnostic precision in orthodontics and radiology. The model performed consistently for both unilateral and bilateral impactions. It reliably identified the position of the maxillary canine relative to nearby teeth, the sinus floor, and the alveolar ridge. The panoramic model showed slightly lower recall for bilateral impactions, which was expected. Symmetrical patterns in 2D images often lead to greater overlap, making it harder to judge positions. The CBCT model performed better, with precision and recall values close to 1.0. This shows the benefits of 3D data. Still, the strong agreement between panoramic predictions and CBCT results suggests that important orientation details can often be inferred from 2D images.

The model’s reliable localization performance, verified by expert review, indicates that few-shot models can achieve accurate results with minimal annotated examples [28]. This is particularly relevant in dental radiology, where annotating bounding boxes is labor-intensive and difficult to scale. The model consistently identified affected canine crowns and follicles, demonstrating its ability to handle the variability observed in pediatric and adolescent panoramic radiographs. The cross-modality model represents a significant methodological and clinical advancement, learning CBCT categories from 2D panoramic images. Clinicians can use this AI tool to support initial diagnoses, especially when access to advanced imaging is limited or when minimizing radiation exposure is important. The tool converts complex 3D diagnostic data from standard radiographs into useful insights. Its ability to detect palatal and combined impactions, even when features are subtle in 2D images, shows that it can identify certain clinical patterns reliably.

Our results are similar to and build on earlier studies. For panoramic classification, Abdulkreem et al. reported an accuracy of approximately 96% on cropped images of impacted canines [29]. Aljabri et al. achieved 92.6% accuracy using deep CNNs [16]. These results are close to the 94% accuracy reported. This suggests that classifying whole images with MobileNetV2 and transfer learning can match, or sometimes outperform, models that use only cropped regions.

Localization results contrast sharply with earlier studies. Özcan et al. reported only about 68% accuracy in simple 2D localization from panoramic radiographs [30]. Minhas et al. saw modest pseudo-3D predictive accuracy (about 41% buccolingual; 55% mesiodistal) [31]. In contrast, our few-shot Faster R-CNN localization achieved 98% accuracy when validated by experts. The use of transfer learning, careful augmentation, and a two-stage detection strategy seems to address many of the limitations seen in these earlier works. Research on root resorption also shows the benefits of cross-modality learning. Alqerban et al. found moderate accuracy in predicting resorption risk from panoramic images alone (AUC about 0.74 overall, 0.80 for severe cases) [17]. In contrast, CBCT-based methods, such as those reported by de Araujo et al., achieved higher accuracy (AUC of about 0.89–0.91) [32]. Our study supports the use of panoramic X-rays for screening. However, CBCT is still best for subtle or complex 3D diagnoses.

Finally, classic work by Ericson and Kurol emphasized the inherent limitations of panoramic radiographs, distortion, magnification, overlapping structures, and unreliable buccopalatal information [33]. The small yet consistent performance gap between panoramic AI predictions and CBCT results is fully consistent with these foundational observations. However, the cross-modality model reached κ = 0.91. This result shows that deep learning can extract substantial anatomical information from panoramic projections, helping mitigate some of the model’s limitations.

These results support the use of AI for diagnosis in orthodontic practice, in accordance with ALARA principles. This framework can be interpreted as a structured AI-assisted diagnostic protocol for the evaluation of impacted maxillary canines using panoramic radiographs. For routine pediatric and teen cases, panoramic radiographs with AI-based analysis in the near future might help to identify impaction, evaluate severity, and inform early treatment decisions. The model can flag unusual paths, asymmetries, or proximity to roots to help triage cases in busy clinics. The AI system functions as a decision support tool, intended to assist rather than replace clinical judgment. When a panoramic image is unclear or presents high-risk features, CBCT remains necessary for definitive evaluation. For general practitioners, AI-enabled interpretation seeks to enhance diagnostic consistency, facilitate timely referrals, and support clinical decision-making in complex impaction cases. In settings where CBCT access is limited or cost-prohibitive, the cross-modality model enables estimation of three-dimensional canine position using only panoramic radiographs. This approach increases the clinical utility of standard imaging by improving triage and identifying cases that require higher-resolution scans, thereby supporting clinics with limited resources or operating in mobile environments without advanced imaging capabilities.

Recent digital health research emphasizes that the use of AI in diagnostic processes requires careful consideration of transparency, data protection, and patient comprehension [34,35]. Patients should be informed when AI is used to interpret radiographs, particularly when it affects decisions about additional imaging, such as CBCT. Although routine image processing may fall under implied consent, the application of advanced algorithms may necessitate updated patient information and explicit communication during the consent process. The treating dentist remains accountable for all clinical decisions. AI model predictions should be accompanied by confidence levels and clearly stated limitations. Clinicians must retain the authority to override AI-generated suggestions. Continuous performance monitoring, local audits, and bias assessments are essential to ensure safe implementation, particularly across varying patient ages, dental development stages, and imaging equipment.

Several methodological strengths are evident in this study. It features a direct comparison with CBCT for both classification and cross-modality evaluation, employs transfer learning and few-shot strategies suitable for small and imbalanced medical datasets, and integrates classification with localization components to enhance interpretability. Several important limitations remain. Data collection from a single center may restrict the generalizability of the findings to other devices, patient populations, and imaging protocols. The quality of panoramic radiographs was inconsistent, particularly for low-dose pediatric images, which could influence the model’s confidence. The system was not evaluated prospectively, nor were real-world clinical outcomes such as changes in CBCT referral rates, diagnostic efficiency, or treatment planning decisions assessed. Additionally, this study did not incorporate demographic variables such as age and sex, consistent with its image-based design. Future studies should evaluate the potential impact of these factors on model performance and generalizability. Future research should focus on validating the model across different centers, populations, and imaging equipment. Larger annotated datasets, especially for localization and segmentation, would enable more robust evaluation and could help models estimate root resorption risk from panoramic images. Using data from multiple views, like lateral cephalograms or intraoral scans, may improve buccopalatal separation. CBCT-supervised segmentation models, such as U-Net, could aid resorption grading and cortical boundary assessment. Federated learning could increase dataset diversity while protecting patient privacy.

## 5. Conclusions

AI-assisted panoramic images can be as accurate as cone-beam CT (CBCT) in finding and describing impacted upper canine teeth. For clinicians, this means they can find issues earlier and help patients sooner. As a first step that uses less radiation, this approach can ensure early checks, reduce unnecessary CBCT scans, and support difficult decision-making, improving diagnosis and patient care. More validation and better features that connect different scan types and clearly show decision paths could make it easier to move from the first visit to a treatment plan.

## Figures and Tables

**Table 1:** Dataset preparation and augmentation for panoramic and CBCT images.

Metric	Panoramic	CBCT
Initial Clinical Samples	641	158
Augmentation Target	500 per class	1000 per class
Final Processed N	1935	5703
Exclusion Rate (%)	3.25%	4.95%

**Table 2:** Composition of the dataset and test subset.

Imaging Modality	Original (*N*)	Augmented (*N*)	Total Test Subset
Panoramic	128	259	387
CBCT	32	1108	1140

*N* represents the number of images. The test set was partitioned before augmentation to ensure no data leakage between the training and testing phases. Augmented values are reported for dataset transparency and robustness analysis only. Primary model evaluation was done on the original test set.

**Table 3:** Performance metrics of the MobileNetV2 model for the classification of maxillary canine impaction types on panoramic X-rays.

Type of Impaction	Precision	Recall	F1-Score	No. of Images
Bilateral	0.95	0.85	0.90	97
Normal	0.99	0.98	0.99	96
Unilateral Left	0.90	0.96	0.93	96
Unilateral Right	0.89	0.96	0.92	96

**Table 4:** Performance metrics of the MobileNetV2 model for the classification of maxillary canine impaction types on CBCT.

Type of Impaction	Precision	Recall	F1-Score	No. of Images
Buccal	0.94	0.98	0.99	190
Buccal and Midalveolar	0.93	0.99	0.96	191
Midalveolar	0.97	0.89	0.94	188
Palatal	0.99	0.99	0.99	191
Palatal and Buccal	0.96	0.99	0.98	190
Palatal and Midalveolar	0.99	0.98	0.96	189

## Data Availability

The data presented in this study are available on request from the corresponding author. The data are not publicly available due to patients privacy.

## Abbreviations

The following abbreviations are used in this manuscript:

AI	Artificial Intelligence
ALARA	As Low as Reasonably Achievable
AUC	Area Under the Receiver Operating Characteristic Curve
CBCT	Cone-Beam Computed Tomography
CI	Confidence Interval
CNN	Convolutional Neural Network
CT	Computed Tomography
FSL	Few-Shot Learning
IoU	Intersection over Union
IRB	Institutional Review Board
OPG	Orthopantomogram
RGB	Red, Green, Blue
SPSS	Statistical Package for the Social Sciences
YOLO	You Only Look Once
